# Connectomics and new approaches for analyzing human brain functional connectivity

**DOI:** 10.1186/s13742-015-0045-x

**Published:** 2015-03-25

**Authors:** R Cameron Craddock, Rosalia L Tungaraza, Michael P Milham

**Affiliations:** 1 Center for Biomedical Imaging and Neuromodulation, Nathan Kline Institute for Psychiatric Research, 140 Old Orangeburg Rd, Orangeburg, 10962 New York USA; 2 Center for the Developing Brain, Child Mind Institute, 445 Park Ave, New York, 10022 New York USA

**Keywords:** Human connectome, Functional MRI, Brain graphs, Open data, Open science

## Abstract

Estimating the functional interactions between brain regions and mapping those connections to corresponding inter-individual differences in cognitive, behavioral and psychiatric domains are central pursuits for understanding the human connectome. The number and complexity of functional interactions within the connectome and the large amounts of data required to study them position functional connectivity research as a “big data” problem. Maximizing the degree to which knowledge about human brain function can be extracted from the connectome will require developing a new generation of neuroimaging analysis algorithms and tools. This review describes several outstanding problems in brain functional connectomics with the goal of engaging researchers from a broad spectrum of data sciences to help solve these problems. Additionally it provides information about open science resources consisting of raw and preprocessed data to help interested researchers get started.

## Introduction

With its new emphasis on collecting larger datasets, data sharing, deep phenotyping, and multimodal integration, neuroimaging has become a data-intensive science. This is particularly true for connectomics^a^ where thousands of brain imaging scans, each consisting of hundreds of observations of thousands of variables, are being collected and openly shared through a combination of grass-roots initiatives (e.g. the 1000 Functional Connectomes Project (FCP) [1], the International Neuroimaging Data-sharing Initiative (INDI) [2]) and large-scale international projects (the Human Connectome Project (HCP) [3,4], the Brainnetome [5], the Human Brain Project in EU known as CONNECT [6], the Pediatric Imaging, Neurocognition and Genetics (PING) Study [7], the Philadelphia Neurodevelopmental Cohort [8], the Brain Genomics Superstruct Project (GSP) [9], the National Database for Autism Research (NDAR) [10], and the Nathan Kline Institute Rockland Sample [11]). Although this deluge of complex data promises to enable the investigation of neuroscientific questions that were previously inaccessible, it is quickly overwhelming the capacity of existing tools and algorithms to extract meaningful information from the data. This combined with a new focus on discovery science is creating a plethora of opportunities for data scientists from a wide range of disciplines such as computer science, engineering, mathematics, statistics, etc., to make substantial contributions to neuroscience. The goal of this review is to describe the state-of-the-art in connectomics research and enumerate opportunities for data scientists to contribute to the field.

The human connectome is a comprehensive map of the brain’s circuitry, which consists of brain areas, their structural connections and their functional interactions. The connectome can be measured with a variety of different imaging techniques, but magnetic resonance imaging (MRI) is the most common in large part due to its near-ubiquity, non-invasiveness, and high spatial resolution [12]. As measured by MRI brain areas are patches of cortex (approximately 1cm^2^ area) [13] containing millions of neurons (calculated from [14]); structural connections are long range fiber tracts that are inferred from the motion of water particles measured by diffusion weighted MRI (dMRI); and functional interactions are inferred from synchronized brain activity measured by functional MRI (fMRI) [15]. Addressing the current state-of-the-art for both functional and structural connectivity is well beyond the scope of a single review. Instead, this review will focus on functional connectivity, which is particularly fast-growing and offers many exciting opportunities for data scientists.

The advent of functional connectivity analyses has popularized the application of discovery science to brain function, which marks a shift in emphasis from hypothesis testing, to supervised and unsupervised methods for learning statistical relationships from the data [1]. Since functional connectivity is inferred from statistical dependencies between physiological measures of brain activity (i.e. correlations between the dependent variables), it can be estimated without an experimental manipulation. Thus, functional connectivity is most commonly estimated from “resting state” fMRI scans, during which the study participant lies quietly and does not perform any experimenter specified tasks; when estimated in this way, it is referred to as intrinsic functional connectivity (iFC) [16]. Once iFC is estimated, data mining techniques can be applied to identify iFC patterns that covary with phenotypes, such as indices of cognitive abilities, personality traits, or disease state, severity, and prognosis, to name a few [17]. In a time dominated by skepticism about the ecological validity of psychiatric diagnoses [18], iFC analyses have become particularly important for identifying subgroups within patient populations by similarity in brain architecture, rather than similarity in symptom profiles. This new emphasis in discovery necessitates a new breed of data analysis tools that are equipped to deal with the issues inherent to functional neuroimaging data.

## Review

### The connectome analysis paradigm

**Figure 1:**
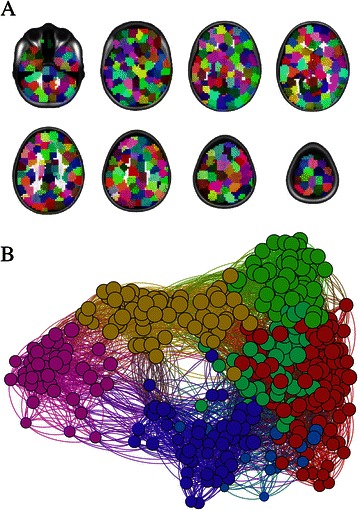
**Parcellation of the brain into functionally homogenous brain regions (A) and the resulting connectome (B).** Community detection identifies seven different modules, which are indicated by the color of the nodes in B.

In 2005 Sporns [19] and Hagmann [20] independently and in parallel coined the term *the human connectome*, which embodies the notion that the set of all connections within the human brain can be represented and understood as graphs. In the context of iFC, graphs provide a mathematical representation of the functional interactions between brain areas: nodes in the graph represent brain areas and edges indicate their functional connectivity (as illustrated in Figure 1). While general graphs can have multiple edges between two nodes, brain graphs tend to be simple graphs with a single undirected edge between pairs of nodes (i.e. the direction of influence between nodes is unknown). Additionally edges in graphs of brain function tend to be weighted - annotated with a value indicating the similarity between nodes. Analyzing functional connectivity involves 1) preprocessing the data to remove confounding variation and to make it comparable across datasets, 2) specification of brain areas to be used as nodes, 3) identification of edges from the iFC between nodes, and 4) analysis of the graph (i.e. the structure and edges) to identify relationships with inter- or intra- individual variability. All of these steps have been well covered in the literature by other reviews [12,17,21] and repeating that information provides little value. Instead we will focus on exciting areas in the functional connectomics literature that we believe provide the greatest opportunities for data scientists in this quickly advancing field.

#### Modeling functional interactions within the connectome

Defining the nodes to use for a connectivity graph is a well described problem that has become an increasingly active area of research [22]. From a neuroscientific perspective there is meaningful spatial variation in brain function that exists at resolutions much finer than what can be measured using modern non-invasive neuroimaging techniques. However, connectivity graphs generated at the spatial resolution of these techniques are too large to be wieldy and there is insufficient fine-grained information about brain function to interpret connectivity results at that level. For that reason, the number of nodes in the connectome is commonly reduced by way of combining voxels into larger brain areas for analysis. This is accomplished using either boundaries derived from anatomical landmarks [23,24], regions containing homogeneous cyto-architecture as determined by post-mortem studies [25], or from clusters determined by applying unsupervised learning methods to functional data [26,27]. The latter approach tends to be preferred since it is not clear that brain function respects anatomical subdivisions, and similar cells may support very different brain functions [27]. Quite a few clustering approaches have been applied to the problem of parcellating brain data into functionally homogenous brain areas, each varying in terms of the constraints they impose on the clustering solution [22,26-31]. The literature provides some evidence that hierarchical clustering based methods perform best [22,28], but no single clustering level has emerged as optimal. Instead, it appears as though there is a range of suitable clustering solutions from which to choose [22,27].

Once the nodes of a connectivity graph have been chosen, the functional connectivity between them is estimated from statistical dependencies between their time courses of brain activity. Although a variety of bivariate and multivariate methods have been proposed for this purpose [17,32], there is a lot of room for new techniques that provide better estimates of the dependencies, or provide more information about the nature of these dependencies. iFC is most commonly inferred using bivariate tests for statistical dependence, typically Pearson’s correlation coefficient [16]. Since these methods only consider two brain areas at the time, they cannot differentiate between direct and indirect relationships. For example the connection *A*⇔*C* in the triangle *A*⇔*B*, *B*⇔*C*, *A*⇔*C* may be due to the variance that *A* and *C* both share with *B* (an indirect connection), rather than variance that is shared uniquely by the two independent of *B* (a direct connection). Indirect relationships can be excluded from the graph using partial correlation, or inverse covariance matrix estimation, but regularization estimators must be employed for large number of brain areas [17,33].

Tests of statistical dependencies between brain regions only provide information about whether or not two nodes are connected, but it should be possible to construct a more precise mathematical description of the relationship between brain areas [34]. Several different modeling techniques have been proposed to this end. Model confirmatory approaches such as structural equation modeling (SEM) [35] and dynamic causal modeling (DCM) [36] can offer fairly detailed descriptions of node relationships, but, they rely on the pre-specification of a model and are limited in the size of network that can be modeled. Cross-validation methods have been proposed to systematically search for the best model [37-39], but simulations have shown that those methods do not necessarily converge to the correct model [40]. Granger causality is another exploratory, data-driven modeling technique that has been particularly popular due to its promise of identifying causal relationships between nodes based on temporal lags between them [41]. However, the assumptions underlying Granger causality do not quite fit with fMRI data [32], where delays in the time-courses between regions may be more reflective of some physiological phenomena, such as a perfusion deficit [42], rather than causal relationships between brain areas. Alternatively, brain connectivity can be inferred from a multivariate regression that is solved using either dimensionality reduction [34] or regularization [43]. These more precise mathematical models of connectivity have shown great promise for testing hypotheses of brain organization [43], predicting response to rehabilitation after stroke data [44], and as biomarkers of disease [45].

Functional interactions within the connectome are commonly considered to be static over the course of an imaging experiment, but a growing body of research has demonstrated that connectivity between brain regions changes dynamically over time [46]. While most studies have measured connectivity within a short window of the fMRI time-course that is moved forward along time [47-50] other methods have been employed with similar results [51,52]. Several problems must be overcome in order to reliably measure changing functional connectivity patterns from the inherently slow and poorly sampled fMRI signal. First, the variance of correlation estimates increases with decreasing window size, meaning that unless proper statistical controls are utilized, the observed dynamics may arise solely from the increased variance [53]. This issue may be mitigated using the new higher speed imaging methods, which have already shown promise for extracting dynamic network modes using temporal independent component analysis (tICA), although large numbers of observations are still necessary [52]. Node definition is another issue, as it is unclear whether brain areas defined from static iFC are appropriate for dynamic iFC; however, initial work has shown that parcellations of at least some brain regions from dynamic iFC are consistent with what is found with static [49].

#### Mapping intra- and inter-individual variation

The ultimate goal of connectomics is to map the brain’s functional architecture and to annotate it with the cognitive or behavioral functions they subtend. This latter pursuit is achieved by a group level analysis in which variations in the connectome are mapped to inter-individual differences in phenotype [21], clinical diagnosis [54], or intra-individual responses to experimental perturbations (such as the performance of different tasks) [55-57]. Several different analyses have been proposed for accomplishing these goals, and they all require some mechanism for comparing brain graphs [17].

Approaches to comparing brain graphs can be differentiated based on how they treat the statistical relationships between edges. One such approach, referred to as "bag of edges", is to treat each edge in the brain graph as a sample from some random variable. Thus, a set of *N* brain graphs each with *M* edges will have *N* observations for each of the *M* random variables. In this case, the adjacency (or similarity) matrix describing the brain graphs can be flattened into a vector representation and any of the well explored similarity or dissimilarity metrics can be applied to the data [12]. One of the benefits of this representation is the ability to treat each edge as independent of all other edges and to compare graphs using mass univariate analysis, in which a separate univariate statistical test (e.g. t-test, anova, or ancova) is performed at each edge. This will result in a very large number of comparisons and an appropriate correction for multiple comparisons, such as Network-Based Statistic [58], Spatial Pairwise Clustering [58], Statistical Parametric Networks [59], or group-wise false discovery rate [60], must be employed to control the number of false positives. Alternatively, the interdependencies between edges can be modeled at the node level using multivariate distance matrix regression (MDMR) [61], or across all edges using machine learning methods [62-64].

Despite the successful application of this technique, a drawback of representing a brain graph as a bag of edges is that it throws away all information about the structure of the graph. Alternative methods such as Frequent Subgraph Mining (FSM) rely on graph structure to discover features that better discriminate between different groups of graphs [65]. For instance, Bogdanov *et al.* [66] were able to identify functional connectivity subgraphs with a high predictive power for high versus low learners of motor tasks. A recent comprehensive review [67] outlines other approaches that take the graph structure into account e.g. the graph edit distance and a number of different graph kernels. All of these methods are under active development and have not yet been widely adapted by the connectomics community.

Another approach for estimating graph similarity using all the vertices involves computing a set of *graph-invariants* such as node centrality, modality, and global efficiency, among others, and using the values of these measures to represent the graph [68,69]. Depending on the invariant used, this approach may permit the direct comparison of graphs that are not aligned. Another advantage is that invariants substantially reduce the dimensionality of the graph comparison problem. On the other hand, representing the graph using its computed invariants throws away information about that graph’s vertex labels [70]. Moreover, after computing these invariants it is often unclear how they can be interpreted biologically. It is important that the invariant used matches the relationships represented by the graph. Since edges in functional brain graphs represent statistical dependencies between nodes and not anatomical connections, many of the path-based invariants do not make sense, as indirect relationships are not interpretable [68]. For example, the relationships *A*⇔*B* and *B*⇔*C* do not imply that there is a path between nodes *A* and *C*; if a statistical relationship between *A* and *C* were to exist they would be connected directly.

##### Predictive modeling

Resting state fMRI and iFC analyses are commonly applied to the study of clinical disorders and, to this end, the ultimate goal is the identification of biomarkers of disease state, severity, and prognosis [54]. Prediction modeling has become a popular analysis method because it most directly addresses the question of biomarker efficacy [62,63,67]. Additionally, the prediction framework provides a principled means for validating multivariate models that more accurately deal with the statistical dependencies between edges compared to mass univariate techniques, all while reducing the need to correct for multiple comparisons.

The general predictive framework involves learning a relationship between a *training* set of brain graphs and a corresponding categorical or continuous variable. Brain graphs can be represented by any of the previously discussed features. The learned model is then applied to an independent *testing* set of brain graphs to decode or *predict* their corresponding value of the variable. These values are compared to their "true" values to estimate *prediction accuracy* - a measure of how well the model generalizes to new data. Several different strategies can be employed to split the data into training and testing datasets, although leave-one-out cross-validation has high variance and should be avoided [71].

A variety of different machine learning algorithms has been applied to the analysis of brain graphs in this manner, but by far the most commonly employed has been support vector machines [54,72]. Although these methods offer excellent prediction accuracy, they are often black boxes, for which the information used to make the predictions is not easily discernible. The extraction of neuroscientifically meaningful information from the learned model can be achieved by employing sparse methods [73] and feature selection methods [62] to reduce the input variables to only those essential for prediction [17]. There is still considerable work to be performed in 1) improving the extraction of information from these models, 2) developing techniques permitting multiple labels to be considered jointly, and 3) developing kernels for measuring distances between graphs.

There are a few common analytical and experimental details that limit the utility of putative biomarkers learned through predictive modeling analyses. Generalization ability is most commonly used to measure the quality of predictive models. However, since this measure does not consider the prevalence of the disorder in the population, it does not provide an accurate picture of how well a clinical diagnostic test based on the model would perform. This can be obtained from estimates of positive and negative predictive values [74,75] using disease prevalence information from resources such as Centers for Disease Control and Prevention Mortality and Morbidity Weekly Reports [76]. Castellanos *et al.* provide a reevaluation of generalizability metrics reported in the connectomics prediction literature up to 2013. Also, the majority of neuroimaging studies are designed to differentiate between an ultra-healthy cohort and a single severely-ill population, which further waters down estimates of specificity. Instead, it is also important to validate a biomarker’s ability to differentiate between several different disease populations - an understudied area of connectomes research [18].

Most predictive modeling-based explorations of connectomes have utilized classification methods that are sensitive to noisy labels. This is particularly problematic given the growing uncertainty about the biological validity of classical categorizations of mental health disorders [18]. This necessitates the use of methods that are robust to noisy labels [77,78]. Many such techniques require quantification of the uncertainty of each training example’s label, which can be very difficult to estimate for clinical classifications. Another approach that is being embraced by the psychiatric community is to abandon classification approaches altogether, and to instead focus on dimensional measures of symptoms [79]. In the context of predictive modeling this translates into a change in focus toward regression models, which to date have been underutilized for the analysis of connectomes [54].

**Figure 2:**
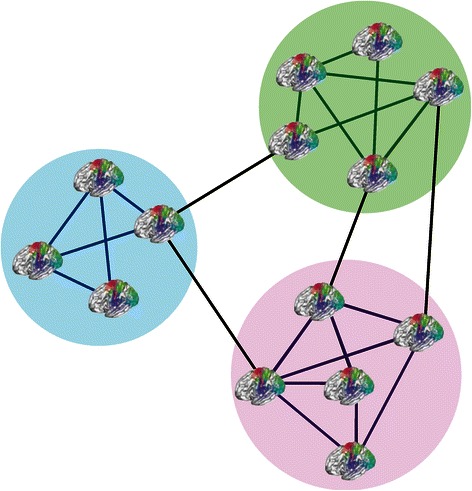
**Identifying communities based on neurophenotypes. Brain glyphs provide succinct representations of whole brain functional connectivity [** 85 **].**

The aforementioned dissatisfaction with extant clinical categories opens up opportunities to redefine clinical populations based on their biology rather than symptomatology. This can be accomplished using unsupervised learning techniques to identify subpopulations of individuals based on indices of brain function and then identifying their associated phenotypes, as illustrated in Figure 2 [80]. Similar to predictive modeling, a major challenge of this approach is to find the features that are most important for defining groups. Another problem is regularizing the clustering solution to make sure it is relevant to the phenotypes under evaluation. These issues can be resolved using semi-supervised techniques or "multi-way" methods that incorporate phenotypic information to guide clustering [81]. Along these lines, joint- or linked- ICA methods have been used to fuse different imaging modalities [82,83] as well as genetics and EEG data with imaging data [84].

#### Evaluating functional connectivity pipelines

Analyzing functional connectivity data requires the investigator to make a series of decisions that will impact the analysis results; examples include choosing the preprocessing strategy for removing noise, the parcellation method and scale for defining graph nodes, the measure for defining connectivity, and the features and methods for comparing connectivity across participants. Several different possibilities have been proposed for each of these steps and choosing the best analysis strategy is a critical problem for connectome researchers. The complexity of this problem is highlighted by observations that both uncorrected noise sources [86-90] and denoising strategies [91,92] can introduce artifactual findings. Ideally the choices for each of these parameters would be determined by maximizing the ability of the analysis to replicate some ground truth, but - as with most biomedical research - the ground truth is unknown. Simulations provide useful means for comparing the performance of different algorithms and parameter settings, but are limited by the same lack of knowledge that necessitates their use. Instead researchers are forced to rely on criteria such as prediction accuracy, reliability, reproducibility, and others for model selection [93]. Although most published evaluations of different connectivity analysis strategies focus on single optimization criterion in isolation, doing so may result in a sub-optimal choice. For example, head motion has high test-retest reliability, as do the artifacts that are induced by head motion [89]. As such, focusing solely on test-retest reliability may lead to the conclusion that motion correction should not be employed. Likewise, when learning a classifier for a hyperkinetic population, head motion-induced artifacts will improve prediction accuracy [94]. Instead, several - ideally orthogonal - metrics should be combined for model selection. For example, in the case of motion correction, metrics for model selection should include an estimate of residual head motion effects in the data [87-90]. Failure to include measures of prediction accuracy and reproducibility in the optimization might result in a strategy that is too aggressive and removes biological signal [95,96]. Going forward, the development of new frameworks and metrics for determining the best algorithms for connectivity analysis will continue to be a crucial area of research.

### Computational considerations

Many of the advances in connectomics research have been spurred on by Moore’s Law and the resulting rapid increase in the power and availability of computational resources. However, the amount of resources, time and memory required to process and analyze large connectomics datasets remains a significant barrier for many would-be connectomes researchers, hence providing another crucial area where computational researchers can contribute to connectomics research. The most common approach for automating high-throughput connectomics processing is to link existing neuroimaging tools together into software *pipelines*. In most cases, since processing each dataset can be performed independently, these pipelines can be executed in parallel on large-scale, high-performance computing (HPC) architectures, such as multi-core workstations or multi-workstation clusters [97-102]. The construction of these pipelines are made possible by the modularity of most neuroimaging packages (e.g., AFNI [103], ANTs [104], FSL [105], and SPM [106]), in which each processing step is implemented by separate functionality, and by their reliance on the NIfTI standard [107], which allows tools from different packages to be inter-mixed. Some steps of the pipeline are independent as well, and many of the toolsets are multithreaded, providing further opportunities to speedup processing by taking advantage of multi-core systems. Using this strategy, the execution time for a large-scale analysis can theoretically be sped up by the number of pipelines that are run in parallel, but in practice this is not quite obtainable due to overheads incurred by the increased competition for resources (Amdahl’s Law [108]). A major advantage of this strategy is that no modifications to the existing neuroimaging tools are required, plus it can be easily scaled to very large datasets, and it can take advantage of everything from relatively small multi-core systems to very large computing clusters. A disadvantage is that it requires access to large computational resources that are not always available, particularly at smaller research institutions, or in developing countries.

Since the preprocessing and analysis of large connectomics datasets are bursty in nature, they do not justify the large capital costs and maintenance burden of dedicated HPC infrastructures [109]. Instead, when shared or institutional computing resources are unavailable, cloud computing offers a “pay as you go” model that might be an economical alternative. Catalyzed by virtualization technology, systems such as the Amazon Elastic Compute Cloud and Google Compute Engine allow users to dynamically provision custom-configured HPC systems to perform an analysis. Pre-configured virtual machines such as the Configurable Pipeline for the Analysis of Connectomes Amazon Machine interface (C-PAC AMI) [110] and the NITRC Computational Environment (NITRIC-CE) [111] eliminate many of the challenges associated with installing and maintaining open source tools. Preprocessing a single dataset (structural MRI and functional MRI for a single participant) using the C-PAC AMI costs around $2.50 on Amazon EC2 using computation-optimized compute nodes with 32 processors and 60 gigabytes of RAM, this could cost as little as $0.75 per dataset if more economical “spot” instances are utilized. The largest drawbacks to computing in the cloud are the time required for data transfers and the expense.

The previously described strategies for accelerating functional connectivity analyses rely on the data parallelism that exists between datasets, but there is quite a bit of parallelism that exists at the voxel level that can be exploited using graphics processing unit (GPU) architectures [112]. It is well established that GPU computing systems can achieve similar computation throughputs (floating point operations per second; FLOPS) as computing clusters, using less expensive equipment and less power [112,113]. Currently, tools that offer GPU implementations are BROCCOLI [114], freesurfer [115] and FSL [116]. Compared to the fastest multi-threaded implementation, BROCCOLI has achieved 195× speedup for nonlinear registration and is 33× faster for permutation testing [117]; the GPU implementation of freesurfer achieves a 6× increase in speed for cortical extraction [115]; a GPU implementation achieved 100× speedup for diffusion tractography [116]; and experiments with calculating functional connectivity using GPUs found a mean increase of 250× more speed over a CPU implementation [118]. The speedups for permutation testing enable more accurate tests of statistical significance, as well as the objective comparison of statistical methods [119]. For example, the increase in speed afforded by GPUs made it possible to perform an in-depth evaluation of the specificity of statistical parameter mapping for task fMRI analyses in ten days; a simulation that would have taken 100 years on standard processors [120]. The major drawbacks of using GPUs for connectomes analysis are that few tools have been ported to these architectures and the additional level of programming sophistication required to develop software for GPUs, although programming libraries such as OpenCL (e.g. as described in Munshi *et al.* [121]) are simplifying the latter.

### Open science resources for big data research

Significant barriers exists for “big data” scientists who wish to engage in connectomics research. The aforementioned imaging repositories have allowed significant progress to be made in assembling and openly sharing large datasets comprised of high-quality data from well-characterized populations. Before a dataset can be analyzed it must be preprocessed to remove nuisance variation and to make it comparable across individuals [93]. Additionally, the quality of the data must be assessed to determine if they are suitable for analysis. Both of these are daunting chores, and although several open source toolsets are available for performing these tasks, they require a significant amount of domain-specific knowledge and labor to accomplish. The Preprocessed Connectomes Project (PCP) [122], the Human Connectome Project (HCP) [3,4], and others, are directly addressing this challenge by sharing data in its preprocessed form. The biggest challenge faced by these preprocessing initiatives is determining the preprocessing pipeline to be implemented. The HCP takes advantage of the uniformity its data collection to choose a single optimized pipeline [123]. Favoring plurality, the PCP approaches this problem by preprocessing the data using a variety of different processing tools and strategies. After an analysis is complete, the results can be compared to previous results from other analyses to assess their validity and to assist in their interpretation. Several hand-curated and automatically generated databases of neuroimaging results exist to aide in this effort [124-127]. Several data-sharing resources for raw and preprocessed neuroimaging data are listed in Section “List of resources for openly shared raw and processed neuroimaging data”; a nearly comprehensive index of open source software packages for working with neuroimaging data can be found at the Neuroimaging Informatics Tools and Resources Clearinghouse (NITRC) [128].

### List of resources for openly shared raw and processed neuroimaging data

- http://fcon_1000.projects.nitrc.org 1000 Functional Connectomes (FCP) ^⋆^: Raw resting state functional MRI and structural MRI for more than 1200 healthy individuals from 33 different contributors [1].
- https://thedata.harvard.edu/dvn/dv/GSP Brain Genomics Superstruct Project (GSP) ^⋆^: Raw resting state functional MRI, and structural MRI data, along with automated quality assessment and pre-computed brain morphometrics, and cognitive, personality, and behavior data for 1570 healthy, college-age individuals (18-35 years old) acquired using one of four MRI scanners. 1139 of the participants have second resting-state fMRI scans acquired from the same scanning session, and 69 have re-test scans [9].
- http://fcon_1000.projects.nitrc.org International Neuroimaging Datasharing Initiative (INDI): A follow-up to the 1000 Functional Connectomes Project, which shares raw resting state functional MRI, task-based functional MRI, structural MRI, and diffusion MRI data for 20 different projects; nine of which are being shared prospectively, as they are collected, and before publication. INDI contains data from a variety of different clinical populations and other experimental designs [2]. Notable examples are the http://fcon_1000.projects.nitrc.org/indi/adhd200 ADHD-200 [129], which contains 490 individuals with ADHD and 598 typically developing controls, the http://fcon_1000.projects.nitrc.org/indi/abideAutism Brain Imaging Data Exchange (ABIDE; 539 Autism and 573 healthy controls) [130], the http://fcon_1000.projects.nitrc.org/indi/CoRR/html/ Consortium for Reliability and Reproducibility (CoRR) [131], which contains test-retest datasets on over 1600 individuals, and the http://fcon_1000.projects.nitrc.org/indi/enhanced/ Enhanced Nathan Kline Institute-Rockland Sample [11], which is a community ascertained longitudinal sample with deep phenotyping.
- http://www.humanconnectomeproject.org/ Human Connectome Project (HCP): Raw and preprocessed resting state functional MRI, task functional MRI, structural MRI, diffusion MRI, deep phenotyping, and genetics data collected from a variety of individuals, including 1200 healthy adults (twins and non-twin siblings) by two consortia: one between Washington University St. Louis and University of Minnesota [4] and another between Massachusetts General Hospital and the University of Southern California [3]. The connectome projects are also developing and sharing imaging analysis pipelines and toolsets.
- http://ndar.nih.gov/ National Database for Autism Research (NDAR) ^⋆^: An NIH-funded data repository of raw and preprocessed neuroimaging, phenotypic, and genomic data from a variety of different autism experiments [10].
- https://openfmri.org/ OpenFMRI: Raw and preprocessed data along with behavioral data for a variety of different task-based functional MRI experiments [132].
- http://pingstudy.ucsd.edu/ Pediatric Imaging, Neurocognition and Genetics (PING) Study: A multisite project that has collected “neurodevelopmental histories, information about mental and emotional functions, multimodal brain imaging data and genotypes for well over 1000 children and adolescents between the ages 3 and 20” [7]. Preprocessed structural and diffusion MRI data are also shared.
- http://www.med.upenn.edu/bbl/projects/pnc/PhiladelphiaNeurodevelopmentalCohort.shtml Philadelphia Neurodevelopmental Cohort: Raw structural MRI, diffusion MRI, task functional MRI, resting state fMRI, cerebral blood flow, neuropsychiatric assessment, genotyping, and computerized neurocognitive testing data for 1445 individuals, 8-21 years old, including healthy controls and individuals with a variety of diagnoses [8].
- http://preprocessed-connectomes-project.github.io/ Preprocessed Connectomes Project (PCP) ^⋆^: Preprocessed data, common statistical derivatives, and automated quality assessment measures for resting state fMRI, structural MRI, and diffusion MRI scans for data shared through INDI [122].

^⋆^These repositories contain data that is also available in INDI.

## Conclusion

Functional connectomics is a “big data” science. As highlighted in this review, the challenge of learning statistical relationships between very high dimensional feature spaces and noisy or underspecified labels is rapidly emerging as rate-limiting steps for this burgeoning field and its promises to transform clinical knowledge. Accelerating the pace of discovery in functional connectivity research will require attracting data science researchers to develop new tools and techniques to address these challenges. It is our hope that recent augmentation of open science data-sharing initiatives with preprocessing efforts will catalyze the involvement of these researchers by reducing common barriers of entry.

## Endnote

^a^ Consistent with the literature, we use the term connectome to refer to the sum total of all connections in the human brain, and connectomics to refer to the scientific field dedicated to studying these connections.
